# Transcriptomic Profiling of Virus-Host Cell Interactions following Chicken Anaemia Virus (CAV) Infection in an *In Vivo* Model

**DOI:** 10.1371/journal.pone.0134866

**Published:** 2015-08-05

**Authors:** Efstathios S. Giotis, Lisa Rothwell, Alistair Scott, Tuanjun Hu, Richard Talbot, Daniel Todd, David W. Burt, Elizabeth J. Glass, Pete Kaiser

**Affiliations:** 1 Agri-Food and Biosciences Institute, Belfast, United Kingdom; 2 Queen’s University Belfast, Belfast, United Kingdom; 3 The Roslin Institute and R(D)SVS, University of Edinburgh, Edinburgh, United Kingdom; 4 Institute for Animal Health, Compton, United Kingdom; University of California, Davis, UNITED STATES

## Abstract

Chicken Anaemia Virus (CAV) is an economically important virus that targets lymphoid and erythroblastoid progenitor cells leading to immunosuppression. This study aimed to investigate the interplay between viral infection and the host’s immune response to better understand the pathways that lead to CAV-induced immunosuppression. To mimic vertical transmission of CAV in the absence of maternally-derived antibody, day-old chicks were infected and their responses measured at various time-points post-infection by qRT-PCR and gene expression microarrays. The kinetics of mRNA expression levels of signature cytokines of innate and adaptive immune responses were determined by qRT-PCR. The global gene expression profiles of mock-infected (control) and CAV-infected chickens at 14 dpi were also compared using a chicken immune-related 5K microarray. Although in the thymus there was evidence of induction of an innate immune response following CAV infection, this was limited in magnitude. There was little evidence of a Th1 adaptive immune response in any lymphoid tissue, as would normally be expected in response to viral infection. Most cytokines associated with Th1, Th2 or Treg subsets were down-regulated, except IL-2, IL-13, IL-10 and IFNγ, which were all up-regulated in thymus and bone marrow. From the microarray studies, genes that exhibited significant (greater than 1.5-fold, false discovery rate <0.05) changes in expression in thymus and bone marrow on CAV infection were mainly associated with T-cell receptor signalling, immune response, transcriptional regulation, intracellular signalling and regulation of apoptosis. Expression levels of a number of adaptor proteins, such as src-like adaptor protein (SLA), a negative regulator of T-cell receptor signalling and the transcription factor Special AT-rich Binding Protein 1 (SATB1), were significantly down-regulated by CAV infection, suggesting potential roles for these genes as regulators of viral infection or cell defence. These results extend our understanding of CAV-induced immunosuppression and suggest a global immune dysregulation following CAV infection.

## Introduction

Chicken anaemia virus (CAV) is an immunosuppressive gyrovirus of the Circoviridae family with high prevalence, which causes severe anaemia, haemorrhages and immunosuppression in young chickens [1–4]. Such effects reduce the efficiency of routine vaccinations, while aggravating the effects of other pathogens, causing considerable economic losses to the poultry industry [4–6]. Chickens can be infected with CAV, both vertically and horizontally [7]. Subclinical disease of commercial broilers due to CAV is more common than clinical disease [3].

Vertical transmission causes clinical disease in young chicks at 10–14 days of age, resulting in immunosuppression. Clinical disease is now controlled by ensuring that breeder flocks have sero-converted before they come into lay, either by natural infection or vaccination. Protection of the newly hatched chick is therefore through maternally derived antibody (MDA). Once MDA has gone (2–3 weeks post hatch), CAV can also infect older birds by horizontal transmission resulting mainly in sub-clinical disease. Sub-clinical disease has economic, health and welfare consequences, leading to lower income, higher feed conversion ratios and lower body weight [8]. Importantly, CAV is increasing in prevalence and infection increases susceptibility to a wide variety of other avian pathogens, both viral and bacterial, presumably through immunosuppression of the CAV-infected bird [5].

CAV is one of the smallest animal viruses and as such, information on its interaction with the host is of intrinsic scientific interest. CAV has an unusually small, circular, single-stranded DNA genome encoding three open reading frames for a single structural protein, VP1 and two non-structural proteins, VP2 and VP3, together with a non-coding region, which contains gene regulatory elements. VP2 is a dual specificity protein tyrosine phosphatase [9] and VP3 (also known as “apoptin”) induces apoptosis in various human tumour and transformed cells [10, 11]. It has been suggested that apoptosis induced by this protein is responsible for the lymphoid depletion caused by CAV infection [12]. Phosphorylation at threonine 108 in VP3 is important for the nuclear localisation and cell killing ability of apoptin [13]. However, the mechanisms of apoptin-induced cell death and the role of VP2 in the infection/replication process are not yet clear.

The CAV virus targets lymphoid and erythroblastoid progenitor cells. CAV-induced apoptosis of the precursor haemocytoblast cells leads to a decrease of erythrocytes, thrombocytes and granulocytes, causing anaemia, haemorrhages and immunosuppression, resulting in an increased prevalence of secondary infections [1, 4, 14]. Other sites of CAV replication are precursor T-cells in the cortex of the thymus, CD8 cells in the spleen, and to a lesser extent, the bursa of Fabricius [1]. Within 3–4 days post-infection (dpi), viral antigens can be isolated in the bone marrow, while maximal T-cell depletion of lymphoid organs and especially depletion of the thymic cortex, takes place at 10–14 dpi [12, 15]. In the embryo model [9], CAV causes thymic depletion, down-regulation of expression levels of proinflammatory cytokine mRNA in the infected thymus (possibly explaining the lack of an inflammatory response to CAV infection) and down-regulation of Major Histocompatibility Complex (MHC) Class I mRNA expression levels, despite moderate up-regulation of type I interferon (IFN) mRNA expression levels (presumably thereby decreasing the infected thymocytes’ ability to present viral antigen to any responding cytotoxic T-cells).

As suggested above, there are many aspects of the pathogenicity of CAV and the host immune response to it, which remain unclear. For example, it is not known how the host responds during the early stages of CAV infection, which specific signalling pathways are involved in its pathogenesis and how the virus exploits/triggers apoptosis. The effect of CAV infection *in vitro* on the transcriptome of MSB1 cells resulted in an initial wave of inflammatory, anti-apoptotic and anti-viral gene expression changes followed by alterations in genes associated with immunosuppression [16]. In this study, we aimed to advance our understanding of the host pathways that result in clinical disease following CAV infection *in vivo*. Day-old, specific-pathogen-free (SPF) chicks were infected with CAV, which mimicked vertical transmission in the absence of MDA. We measured the responses to CAV in two ways. Firstly, we determined the kinetics of mRNA expression levels of signature cytokines of innate and adaptive immune responses at 4, 7, 11 and 14 dpi. There have been several global transcriptomic analyses of other viral infections of chickens [16–21]. However, few host gene expression studies have been conducted so far following *in vivo* infection with CAV and therefore a number of key genes and pathways involved in the pathogenesis of CAV infection may as yet be unidentified. We therefore also compared the gene expression profiles of mock-infected (control) and CAV-infected chickens at 14 dpi using a chicken immune-related 5K gene expression microarray [21].

## Materials and Methods

### Animal study

All animal experiments throughout this study were conducted according to the policy of the federation of European Laboratory Animal Science Associations and the European Convention for the protection of vertebrate animals used for experimental and other scientific purposes, with implementation of the principles of the 3R's (replacement, reduction, refinement). Ethical permission specifically for the experiments described here was obtained from the Queen's University Animal Welfare and Ethics Review Board and all researchers carrying out the work had Personal Licences from the UK Home Office. Animals were housed in negative pressure isolators under standard conditions of temperature and humidity. They were monitored daily and had *ad libitum* access to feed and water. No mortality occurred and no animal met criteria for early euthanasia during the study. At the end of the experiment, birds were weighed, anesthetized by CO_2_ inhalation and euthanized by cervical dislocation. Tissue samples (1g) were collected and placed in RNAlater (Ambion) and stored at 4°C for 24 h followed by long-term storage at −70°C prior to RNA extraction.

White Leghorn (SPF) chickens hatched from eggs from a CAV-free source, shown to be free of maternal antibody to CAV as determined by IIF [22], were used throughout this study. Eight groups of chickens, (*n* = 6 to 9) housed in separate negative-pressure isolators, were either intramuscularly infected at day 1 of age with 0.2 ml of a 106.75 TCID/0.1 ml infective dose of CAV Cux1 isolate (Cuxhaven-1) or mock infected (MSB1 cells, Marek's disease virus transformed lymphoblastoid cell line) as previously described [23–25]. At 4, 7 and 11 dpi, tissues (thymus, spleen, bursa of Fabricius and bone marrow) were removed from five CAV-infected and five mock-infected chickens (at 14dpi six animals were sampled from each group) and duplicate samples taken for microarray and quantitative RT-PCR (qRT-PCR) analyses. A negative control group (4 animals), which were not inoculated, was also included. We have previously observed and confirmed that the sequential changes in the lymphoid tissues and organs that follow CAV infection are highly reproducible, with maximal lymphoid depletion observed at 10–14 days post infection [15, 24]. Gross pathological examination of animal tissues was performed as previously described [23, 26] and the results for day 14 when gross lesions were evident, are presented in Table 1. Confirmation of viral infection was conducted by immunostaining for CAV antigen using thymus sections at day 14 using the MAb 2A9 as previously described [24].

**Table 1:** Pathogenicity evaluations of infected, uninfected and mock-infected animals at day 14 post-infection. Gross pathological examination of the thymuses and bone marrows was performed in a blind manner, and a clinical score was estimated by scoring the severity of the thymus atrophy and paleness of the bone marrow, as 0, 1 or 2.

Group description	No. of chicks	Haematocrit values	Mean haematocrit value ± SD	No. of chicks with anaemia (%)^*a*^	No. of chicks (score) Bone Marrow	No. of chicks (score) Thymus	Mean Clinical Score
Infected^*b*^	9	34, 21, 13, 9, 24, 28, 20, 30, 21	22.22 ± 7.93	7 (78)	4 (1), 5 (2)	7 (1), 1 (2)	2.55
Mock- infected (MSB1 cells)	6	35, 33, 33, 32, 32, 31	32.66 ± 1.37	0(0)	None	None	0.00
Uninoculated	4	35, 34, 32, 31,	33.00 ± 1.82	0(0)	None	None	0.00

*a* Anaemia is defined as a hematocrit value of less than 27.

*b* The six most affected chicks, based on haematocrit values, were used for microarray analysis.

### RNA extraction and cDNA synthesis

Tissues (30 mg) were homogenised using a bead mill (Retsch, Haan, Germany) and QIAshredders (Qiagen, Crawley, UK). RNA was extracted and purified from homogenates using an RNeasy mini kit (Qiagen) following the manufacturer’s protocol. cDNA synthesis reactions were carried out with 2 μg of total RNA, using Superscript Choice system (Life Technologies, Paisley, UK) with an oligo (dT) primer containing a T7 RNA polymerase promoter (Research Genetics, Huntsville, USA). RNA quantity was measured spectrophotometrically and RNA quality was estimated using an Agilent 2100 Bioanalyser (Agilent Technologies, Waldbronn, 6 Germany). Eluted RNA was stored at -70°C prior to use.

### Real-time quantitative RT-PCR

Messenger RNA expression levels were quantified using TaqMan qRT-PCR as described [27–29]. Primer and probe sequences for cytokine, chemokine and 28S RNA (reference gene)-specific amplification and three genes that were used for validation of microarray analysis (SLA, SATB1 and BAK1) are given in Table 2. Probes were all labelled with the fluorescent reporter dye FAM at the 5’-end and the quencher TAMRA at the 3’-end. Real-time qRT-PCR was performed using the TaqMan Fast universal PCR master mix and one-step RT-PCR master mix reagents (Applied Biosystems, Cheshire, UK). Amplification and detection of specific products were performed using the Applied Biosystems 7500 Fast Real-Time PCR System with the following cycle profile: one cycle of 48°C for 30 min and 95°C for 20 s, followed by 40 cycles of 95°C for 3 s and 60°C for 30 s. Results are expressed as 40-Ct, after normalising each sample using the Ct value for the 28S rRNA product for the same sample, as described previously [27–29]. Briefly, to account for variation in sampling and RNA preparation, the individual gene product Ct values were normalised using the Ct value of the 28S rRNA gene product for the same sample. Normalised Ct were calculated using the formula Ct + (Nt'-Ct') * S/S', where Nt' is the mean Ct for 28S RNA among all samples, Ct' is the mean Ct for 28S RNA in the sample and S and S' are the slopes of the regressions of the standard plots for the test mRNA and the 28S RNA, respectively. Results were then expressed as 40-Ct values and converted to fold-difference (Means are presented in Table 3). Analysis of the difference between transcripts derived from mock-infected and infected groups was carried out using a nonparametric test (two-sided Mann-Whitney test) with *P*<0.05 as significant.

**Table 2:** Real-time quantitative RT-PCR probes and primers.

RNA target	Probe/primer sequence (5’-3’)
28S	Probe (FAM)-AGGACCGCTACGGACCTCCACCA-(TAMRA)
	F GGCGAAGCCAGAGGAAACT
	R GACGACCGATTTGCACGTC
IFN-α	Probe (FAM)-CTCAACCGGATCCACCGCTACACC-(TAMRA)
	F GACAGCCAACGCCAAAGC
	R GTCGCTGCTGTCCAAGCATT
IFN-β	Probe (FAM)-TTAGCAGCCCACACACTCCAAAACACTG-(TAMRA)
	F CCTCCAACACCTCTTCAACATG
	R TGGCGTGCGGTCAAT
IFN-γ	Probe (FAM)-TGGCCAAGCTCCCGATGAACGA-(TAMRA)
	F GTGAAGAAGGTGAAAGATATCATGGA
	R GCTTTGCGCTGGATTCTCA
IL-1β	Probe (FAM)-CCACACTGCAGCTGGAGGAAGCC-(TAMRA)
	F GCTCTACATGTCGTGTGTGATGAG
	R TGTCGATGTCCCGCATGA
IL-2	Probe (FAM)-ACTGAGACCCAGGAGTGCACCCAGC-(TAMRA)
	F TTGGAAAATATCAAGAACAAGATTCATC
	R TCCCAGGTAACACTGCAGAGTTT
IL-4	Probe (FAM)-AGCAGCACCTCCCTCAAGGCACC-(TAMRA)
	F AACATGCGTCAGCTCCTGAAT
	R TCTGCTAGGAACTTCTCCATTGAA
IL-6	Probe (FAM)-AGGAGAAATGCCTGACGAAGCTCTCCA-(TAMRA)
	F GCTCGCCGGCTTCGA
	R GGTAGGTCTGAAAGGCGAACAG
IL-10	Probe (FAM)-CGACGATGCGGCGCTGTCA-(TAMRA)
	F CATGCTGCTGGGCCTGAA
	R CGTCTCCTTGATCTGCTTGATG
IL-12α	Probe (FAM)-CCAGCGTCCTCTGCTTCTGCACCTT-(TAMRA)
	F TGGCCGCTGCAAACG
	R ACCTCTTCAAGGGTGCACTCA
IL-12β	Probe (FAM)-CTGAAAAGCTATAAAGAGCCAAGCAAGACGTTCT-(TAMRA)
	F TGGGCAAATGATACGGTCAA
	R CAGAGTAGTTCTTTGCCTCACATTTT
IL-13	Probe (FAM-CATTGCAAGGGACCTGCACTCCTCTG-(TAMRA)
	F CACCCAGGGCATCCAGAA
	R TCCGATCCTTGAAAGCCACTT
CXCLi2	Probe (FAM)-TCTTTACCAGCGTCCTACCTTGCGACA-(TAMRA)
	F GCCCTCCTCCTGGTTTCAG
	R TGGCACCGCAGCTCATT
TGF-β4	Probe (FAM)-ACCCAAAGGTTATATGGCCAACTTCTGCAT-(TAMRA)
	F AGGATCTGCAGTGGAAGTGGAT
	R CCCCGGGTTGTGTTGGT
BAK	Probe (FAM)-CCACCATCGCCTCC-(TAMRA)
	F CCAAGGAGAACGCCTACGAGTA
	R AATGCCGCTGTCGAACAAG
SATB1	Probe (FAM)-ACACCGCCCAGCCGGCCT-(TAMRA)
	F GGCCCTGCACCTCTCATAAG
	R CTCTCCGTAGCAATAGTAGCAGTTTTG
SLA	Probe (FAM)-TGCCTTGAAGACTCGTGA-(TAMRA)
	F CTCCACGGCAAACCTTTCA
	R GACCATCAGCAACTTCTGAGTAGTG

F: forward primer; R: reverse primer.

**Table 3:** Real-time qRT-PCR cytokine mRNA expression levels following CAV infection in different lymphoid tissues.

	Thymus				Bone marrow				Spleen				Bursa of Fabricius			
mRNA/day	4	7	11	14	4	7	11	14	4	7	11	14	4	7	11	14
IL-1β	**3.74** ^1^	**-5.25**	**-6.11**	-	1.21	-2.73	-	-1.78	**-4.81**	**3.24**	-2.43	-	-	-1.39	-	-
IL-6	**4.89**	**-5.15**	**-7.32**	-1.54	-1.19	**-3.74**	-1.29	1.88	**-7.97**	2.90	-2.69	-5.73	1.68	-1.13	-	-1.96
CXCLi2	-1.31	1.62	-	-	-1.41	1.19	-	2.47	-1.74	-1.99	-2.32	-1.94	1.15	-	-8.21	1.21
IFN-α	**3.46**	-2.52	**-4.64**	-1.39	-2.22	-1.85	1.41	2.16	**-9.95**	-	-1.72	-4.90	-	1.36	-2.25	-
IFN-β	**3.28**	-2.94	**-8.29**	-1.84	-2.02	-1.55	1.61	1.91	**-9.57**	-	-1.70	-4.05	-	1.42	-3.11	-
IL-2	-	11.41	8.66	7.24	1.78	18.53	9.70	-3.08	-1.84	-	-2.43	-2.75	-	-	-	-
IL-12α	-	-	-	-	**-7.76**	**-3.66**	-	-	-	1.37	-1.84	-	-	-	-	-
IL-12β	-	-	-	1.69	-1.26	1.30	1.23	**-9.57**	1.16	-1.48	1.31	-1.48	-2.63	**-3.40**	-	1.30
IFN-γ	1.21	2.08	2.17	2.40	1.86	-	**6.20**	-1.25	1.45	-	-1.59	-1.15	1.62	-1.98	-1.89	-1.37
IL-4	2.99	**-3.57**	**-7.14**	-1.87	1.44	-2.64	-1.29	1.42	**-5.31**	2.95	-4.35	-	-	-1.38	-2.09	1.60
IL-13	1.35	1.65	**5.36**	**3.31**	**6.01**	-	**15.21**	-1.43	-1.24	-	-	-	-	-	-	-
IL-10	-	1.65	**4.93**	9.75	1.38	1.15	**6.30**	-1.61	1.30	-	-	1.93	-	-	-	-
TGF-β4	-1.14	-	1.92	1.30	-	1.21	1.83	1.15	-	-	-1.33	-1.18	-1.69	-1.22	2.20	2.55

^1^Mean fold difference between infected and mock-infected birds at each time-point (n = 5 (6 for d 14)); *P*<0.05, positive values are up-regulated in infected birds and negative values are down-regulated in infected birds.—: no significant difference between groups or mRNA not expressed in one or both groups. Changes greater than three-fold are highlighted in bold.

### Microarray experiment

RNA preparation and labelling was done as previously described [21]. Cy3 or Cy5 was incorporated into each RNA sample using the Fairplay labelling kit (Stratagene, La Jolla, CA) and unincorporated dyes were removed by passing through DyeEx columns (Qiagen). Dye swap was used to minimise labelling inefficiencies. Equal proportions of total RNA extracted from all samples, including from CAV-infected and mock-infected birds, were pooled to create an RNA pool for use as the reference sample in this experiment. There were three replicates per tissue sample with the reference sample in the Cy3 channel, and a further three per tissue sample with the reference sample in the Cy5 channel. Labelling efficiency was determined by running 0.5 μl of each sample on a 1% agarose gel and measuring the intensity of fluorescence on a GeneTac LS iv scanner (Genomic Solutions, Huntingdon, UK). Microarray analysis was conducted using a chicken immune-targeted cDNA array containing 5190 immune transcripts printed in duplicate [21]. The clones in the array were derived from normalised cDNA libraries created from immunologically active tissues such as the bursa of Fabricius, spleen, Peyer’s patch and thymus (ArrayExpress Acc. No: A-MEXP-307). Microarray hybridisations were carried out overnight in a GenetAC automated hybridisation system (Genomic Solutions) as previously described [21]. Original microarray data produced in this study have been deposited according to the MIAME guidelines in the public database ArrayExpress (http://www.ebi.ac.uk/microarray-as/ae/) (Acc. No: E-MEXP-1228).

### Gene expression data analysis

Dried slides were scanned in a Scanarray 5000 scanner (GSI Lumonics, Rugby, UK) fitted with Cy3 and Cy5 filters and data were extracted from the slide using BlueFuse software (BlueGnome, Cambridge, UK). Each slide was normalized separately, using all of the log-ratios of Cy5 to Cy3 intensities for the non-control spots. Normalization and analysis was based on the Limma package of Bioconductor [30], with additional plotting from the Bioconductor Marray package, but with modifications of the Limma models at both normalization and analysis stages. The normalization was a two-step process of spatial, then intensity-dependent, bias correction. The spatial bias correction was carried out separately for each 2x2 group of blocks, by subtracting corresponding row and column means (RC correction, excluding control spots) from each data spot [31]. The intensity-dependent bias was removed by (local) block-lowess [32]. The choice of level (global or local) for the two normalization steps was based on the examination of spatial heat diagrams and MA plots for all four possible normalization combinations. The tissue differences between infected and control samples/birds in their log ratios of treatment to reference intensities for each gene, taking means of replicate spots on each microarray slide, were analysed by regression models. The Limma eBayes correction was used to shrink the residual variances of genes towards their approximate median value, giving moderated t-tests for the assessment of differential expression [30].

The Benjamini and Hochberg [33] false discovery rate (FDR: the percentage of genes expected to be wrongly identified as differentially expressed) is the default multiple testing method in Limma package. Genes with a false discovery rate greater than 5% in either set of data were excluded from further analysis. No significantly differentially regulated genes were found in spleen and bone marrow with an FDR <5% and the FDR cut-off was therefore extended to 20% to identify differentially expressed genes in these tissues. The gene expression fold change cut-off was set at the customary low threshold of 1.5 to focus more on biologically significant changes.

### Gene Pathway analysis

Ingenuity Pathway Analysis (IPA) software (Ingenuity Systems, Redwood City, CA) was used to identify biological pathways and functional networks for the identified significantly expressed genes in thymus. Unigene ID gene identifiers corresponding to these genes were imported into the IPA program. Each identifier was mapped to its corresponding gene object in the Ingenuity pathway knowledge base (http://www.ingenuity.com). Differentially expressed genes within significantly altered pathways were consolidated into interconnected functional networks and canonical pathways. A score (*z*) was computed for each functional network, the negative logarithm of the *P* value that indicates the likelihood of the focus genes in a network being found together due to random chance. Scores of *z* >2 were considered significant, using a 99% confidence level. The significances for biological functions or canonical pathways were then assigned to each network by determining a *P* value for the candidate genes in the functions or pathways networks, compared against the whole Ingenuity pathway knowledge base as a reference set (S1 File). Right-tailed Fisher’s exact test was used with *P*<0.05

## Results

### Cytokine production post-CAV infection

Expression levels of transcripts for the signature pro-inflammatory (IL-1β, IL-6 and CXCLi2), type I IFN (IFN-α and IFN-β), Th1 (IL-12α, IL-12β and IFN-γ), Th2 (IL-4 and IL-13) and Treg (IL-10 and TGF-β4) cytokines were measured by qRT-PCR (Table 3). To focus on the most biologically significant results we will concentrate on differences greater than three-fold gene expression. Overall, expression was generally unaltered or down-regulated following infection compared to levels in mock-infected controls except the thymus.

Across the tissues, there was a sustained down-regulation of the innate immune cytokines with the marked exception of 4 dpi in the thymus. CAV infection induced the pro-inflammatory cytokines IL-1β and IL-6 as well as type I IFNs in the infected thymus compared to mock infected thymus and also compared to the other tissues (Table 3). The up-regulation of IL-1β and IL-6 in the thymus was also reflected at 7 dpi in the spleen. The levels of the other pro-inflammatory cytokine measured, CXCLi2, was generally not altered by CAV infection although it was substantially down-regulated at 11 dpi in the bursa of Fabricius. Of the adaptive cytokines, only IL-2 showed a sustained and substantial increase in mRNA expression levels following infection, and only in the thymus and bone marrow. There was little change in the mRNA levels for the Th1 cytokines, IL-12a and IL-12b, except in the bone marrow where levels were reduced following infection. Similarly, the Th2 cytokine, IL-4 was mainly down-regulated across tissues and time. In contrast, the Th1 cytokine, IFN-γ and the Th2 cytokine, IL-13 and Treg cytokine, IL-10, were all up-regulated in thymus and bone marrow at several time points, especially at 7 and 11 dpi in the thymus and bone marrow, with similar kinetics to those seen with IL-2 up-regulation.

### Comparison of transcriptomes of immune tissues from mock-infected and CAV-infected birds, by microarray analysis

This analysis was conducted at 14 dpi when anaemia was gross pathological lesions were evident (Table 1). The use of an immune-focused array has identified relatively subtle tissue-specific host gene expression patterns following CAV infection. In terms of FDR and actual number, most differentially expressed genes were detected in the thymus. A total of 40 genes were differentially expressed in the thymus (S2 File) with transcription of 21 genes increased more than 1.5-fold, and transcription of 19 genes decreased more than 1.5-fold compared with expression levels in mock-infected thymus (see Table 4 for most differentially regulated transcripts in thymus as identified by IPA analysis). Fourteen genes were identified as differentially regulated in the bone marrow and thirteen in the spleen (S2 File). No significantly differentially expressed genes were seen in the samples from the bursa of Fabricius.

**Table 4:** Most differentially regulated transcripts in thymus as identified by IPA microarray analysis.

Symbol	Entrez Gene Name	Fold Change	IPA Networks^a^	Location	Type(s)
MALT1	Mucosa associated lymphoid tissue lymphoma translocation gene 1	7.359	1	Cytoplasm	Peptidase
IGJ	Immunoglobulin J polypeptide, linker protein for immunoglobulin alpha and mu polypeptides	4.845	1	Extracellular Space	Other
UNG	Uracil-DNA glycosylase	3.820	2	Nucleus	Enzyme
CWC15	CWC15 spliceosome-associated protein homolog (S. cerevisiae)	2.829	4	Nucleus	Other
FN1	Fibronectin 1	2.635	1	Extracellular Space	Enzyme
COPE	Coatomer protein complex, subunit epsilon	2.601	2	Cytoplasm	Transporter
NBL1	Neuroblastoma, suppression of tumorigenicity 1	2.384	2	Nucleus	Other
NFE2L2	Nuclear factor (erythroid-derived 2)-like 2	2.133	1	Nucleus	Transcription regulator
BAK1	BCL2-antagonist/killer 1	1.726	1	Cytoplasm	Other
COX6A1	cytochrome c oxidase subunit VIa polypeptide 1	-1.643	2	Cytoplasm	Enzyme
DDT	D-dopachrome tautomerase	-1.950	1	Cytoplasm	Enzyme
C12H9orf89	Chromosome 12 open reading frame, human C9orf89	-1.998	3	Cytoplasm	Other
GRAP2	GRB2-related adaptor protein 2	-2.059	1	Cytoplasm	Other
ACOX1	Acyl-CoA oxidase 1, palmitoyl	-2.110	1	Cytoplasm	Enzyme
PTP4A3	Protein tyrosine phosphatase type IVA, member 3	-2.413	1	Plasma Membrane	Phosphatase
CD2	CD2 molecule	-2.584	1	Plasma Membrane	Transmembrane receptor
CD8A	CD8a molecule	-2.791	1	Plasma Membrane	Other
SATB1	SATB homeobox 1	-3.233	1	Nucleus	Transcription regulator
SLA	Src-like-adaptor	-3.521	1	Plasma Membrane	Other

^a^ Networks identified by Ingenuity pathway Analysis are included in S1 File.

### Differential regulation of genes in response to CAV infection in the thymus

Gene transcripts which were down-regulated (S2 File) included the Src-like adaptor protein (SLA), a negative regulator of TCR signalling, Grb-2-related adapter protein (GRAP2), which is part of the CD28 signalling complex, the CD8α chain precursor and special AT-rich binding protein 1 (SATB1). Genes up-regulated in the thymus (S2 File) included pro-apoptotic Bcl2-antagonist killer 1 (BAK1), mucosa-associated lymphoid tissue lymphoma translocation gene 1 (MALT1), involved in NF-κB activation, fibronectin 1 (FN1), nuclear factor (erythroid-derived 2)-like 2 (NFEF2L2) and genes encoding immunoglobulin chains (IgA, IgG, IgJ LV1L2, IgHV and IgHM).

### Pathway analysis of genes regulated in the thymus

**Fig 1:**
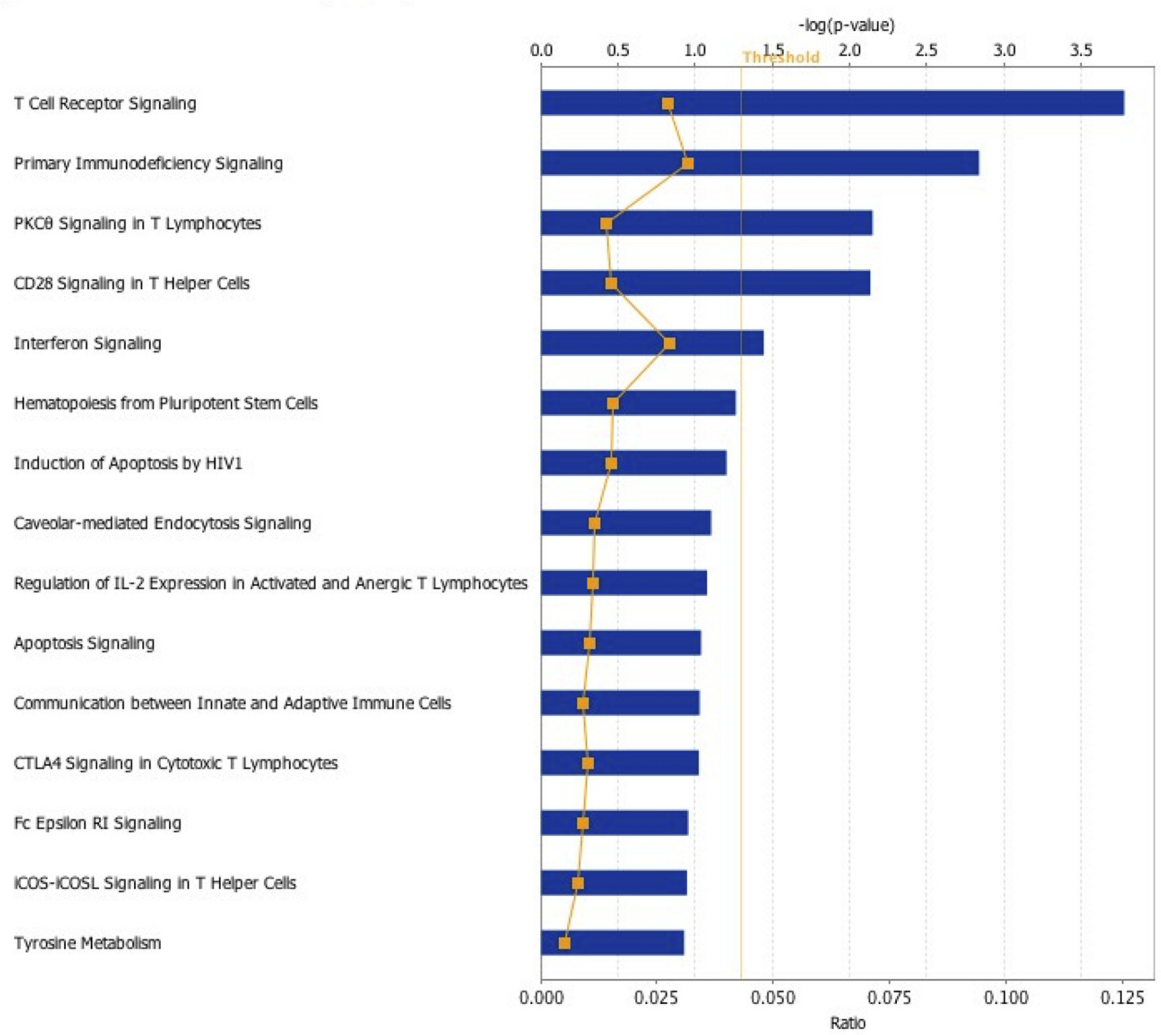
Ingenuity pathway analysis showing the most highly scoring canonical pathways (according to *P* value) associated with the response of thymocytes to CAV infection. Five pathways had a *P* value < 0.05. The orange line represents the ratio of the number of differentially expressed thymus genes in a particular pathway whose expression is correlated with cellular response towards CAV divided by the total number of genes that make up that pathway. Blue bars represent the *P* value for each pathway and are expressed as -1 times the log of the *P* value. The threshold line corresponds to a *P* value of 0.05.

IPA software was used to identify the main host functions and canonical pathways, from the microarray data (S2 File), modulated by CAV infection. Differentially-expressed genes in the thymus fell into diverse functional categories, including cell-cell signalling, immune cell trafficking, inflammatory response, cell death, molecular transport, post-translational modification, cellular growth, development and proliferation (S1 File). Of the 34 differentially modulated canonical pathways that were identified (S1 File), the T-cell receptor (TCR) signalling pathway appeared to be most important for the response of the thymus cells to CAV (*P* value: 1.67E-04, Fig 1).

**Fig 2:**
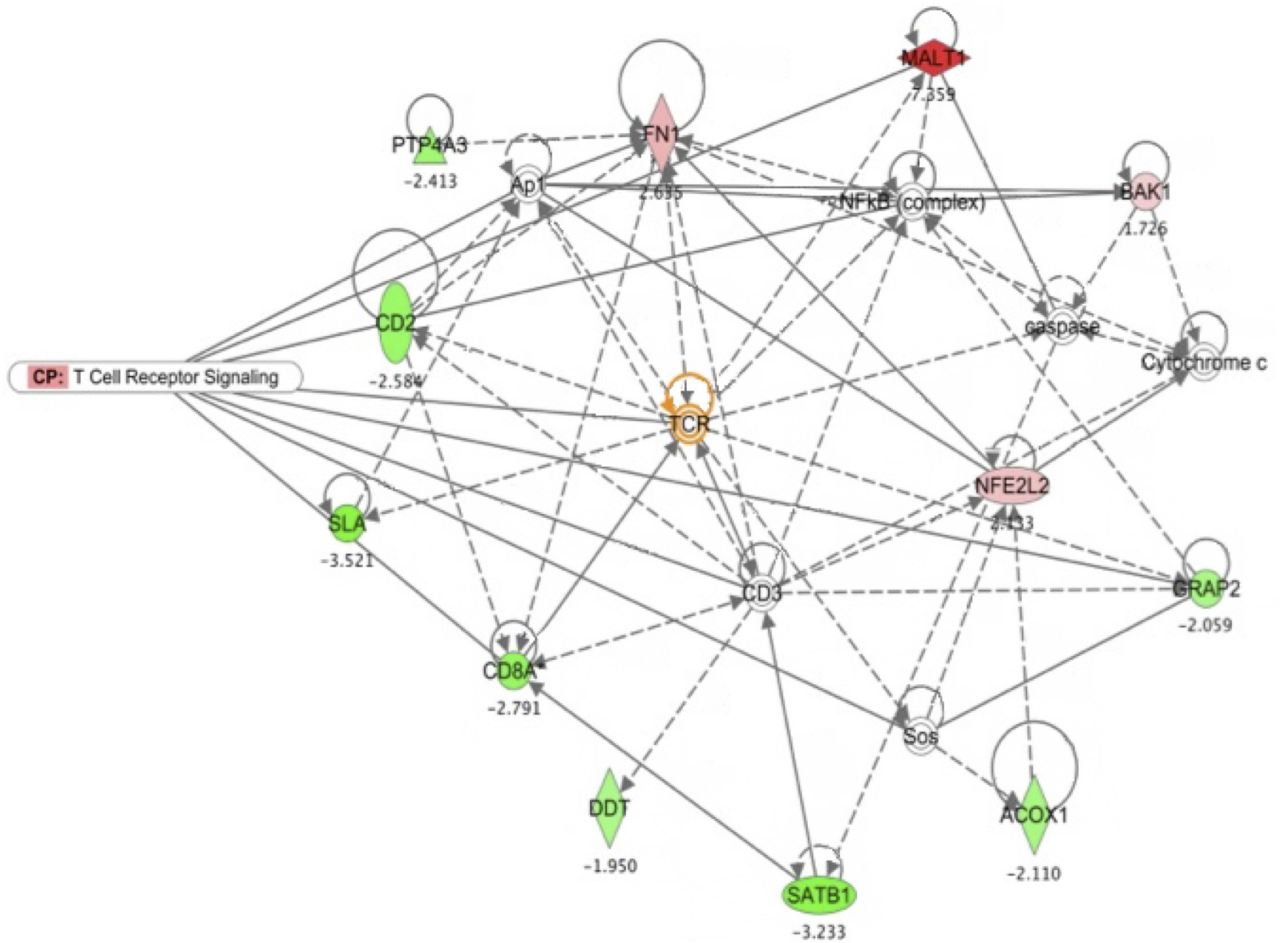
T-cell receptor signalling was the most significant gene network of over-expressed genes in infected samples of thymus as identified by Ingenuity Pathway Analysis (www.ingenuity.com). Colored nodes represent differentially regulated genes (Table 4) while genes in uncolored nodes were not identified as differentially expressed and were integrated into the computationally generated network indicating a relevance to it (S1 File). T-cell receptor signalling gene relationships were overlaid to the network. Solid interconnecting lines represent genes directly connected and dotted lines signify the indirect connection between genes and cellular functions. Top functions of the genes were related to T-cell receptor signalling, immune response and cellular assembly and death, identified around the MALT1 gene (in red). Node shapes represent different biological molecules.

The most significant CAV-induced gene expression changes were observed in the thymus. Analysis of expressed genes in the thymus samples of CAV-infected chickens by IPA identified four gene networks of differentially regulated genes. Gene networks are algorithmically generated based on their connectivity and ranked based upon a score (S1 File). Fig 2 shows the most significant gene network (Score = 33), focused on TCR signalling relationships between differentially regulated genes.

### Validation of microarray data by qRT-PCR

Microarray data were validated by quantifying the mRNA abundance of selected genes using TaqMan qRT-PCR. SLA, BAK1 and SATB1 genes were selected among those significantly regulated and identified by IPA (Fig 2) as significant in CAV infection due to their potential importance in the T-cell receptor signalling and apoptosis pathways. The qRT-PCR (Table 5) was consistent with the microarray data (S2 File) including the direction of regulation i.e., up-regulation or down-regulation, confirming the validity of our data.

**Table 5:** qRT-PCR mRNA expression levels to confirm microarray results following CAV infection in different lymphoid tissues.

	Thymus				Bone marrow				Spleen				Bursa of Fabricius			
mRNA/day	4	7	11	14	4	7	11	14	4	7	11	14	4	7	11	14
SATB1	1.24^1^	-1.62	-2.77	**-7.57**	-	-	-	-	-	-	-	-	-	-	-	-
BAK1	-	1.87	2.34	**-3.46**	-	-	-	-	-	-	-	-	-	-	-	-
SLA	1.55	-1.40	**-3.92**	**-4.28**	-	-	-	-	-	-	-	-	-	-	-1.16	-1.29

^1^Mean fold difference between infected and mock-infected birds at each time-point (n = 5 (6 for d 14)); *P*<0.05, positive values are up-regulated in infected birds and negative values are down-regulated in infected birds.—: no significant difference between groups or mRNA not expressed in one or both groups. Changes greater than three-fold are highlighted in bold.

## Discussion

Hosts typically respond to viral invasion by mounting complex, multifaceted, defensive immune responses that include substantial induction of type I IFNs, initial production of pro-inflammatory cytokines to drive Th1 responses with subsequent induction of the relevant Th1 cytokines, later production of anti-inflammatory cytokines to switch off the inflammatory responses after viral clearance to limit immunopathology and activation of apoptotic pathways. These are all accompanied by notable changes in gene expression. CAV is an immunosuppressive virus, and is thus thought to interfere in at least some of the host antiviral responses. This study is the first global, immune-related analysis of the transcriptional response of host cells to CAV *in vivo*. Microarray analysis was conducted at 14 dpi when anaemia and gross pathological lesions were evident (Table 1) in agreement with previous studies [26, 34]. CAV antigen is first detected in thymus and bone marrow at days 4–7 days post inoculation in 1-day old infected chicks. Subsequently it can be found in other tissues but usually within lymphoid tissue [24]. To investigate the lymph tissue tropism of CAV, we decided to sample the bursa of Fabricius and spleen apart from thymus and bone marrow that are routinely sampled in CAV studies. The most significant CAV-induced gene expression changes among the tissues tested in the study, were observed in the thymus, confirming previous reports that the thymus is the primary target organ for CAV action/infection [35].

Overall, there were no obvious trends in cytokine expression with time that would have been reflective of anti-viral responses in chickens [27, 36, 37]. Interestingly, at 4 dpi in the thymus, there was evidence of induction of an innate immune response, as mRNA expression levels of the pro-inflammatory cytokines IL-1β and IL-6, and the type I IFNs, IFN-α and IFN- β, were up-regulated (between 3- and 5-fold). However, these levels of induction were lower than those seen with other immunosuppressive viral infections, such as infectious bursal disease virus (IBDV) [27] and Marek’s disease virus [37]. For example, following IBDV infection, IL-1β mRNA expression levels were up- regulated 10-fold and those of IL-6, 25-fold. Induced anti-viral innate responses in the chicken normally also involve up-regulation of the IL-8-like chemokine, CXCLi2 [27, 37], but mRNA expression of this chemokine was slightly down-regulated at 4 dpi. Normally, for a viral infection in the chicken (and of course in mammals), such an induced innate response would lead to the induction of a Th1-dominated adaptive immune response, with the production of cytokines such as IL-12 and IFN-γ, from around 5 dpi and peaking between 7 and 14 dpi. However, following CAV infection, IL-12α expression was not up-regulated at all and levels of IFN-γ only increased 2-fold at most. In contrast, up-regulation of IFN-γ by as much as 250-fold was observed following IBDV infection [27] and 25-fold following MDV infection [37]).

However, IL-2 mRNA expression levels were up-regulated from 7–14 dpi, to as high as 11.4-fold, suggesting that there was proliferation of T-cells in response to CAV infection. Further evidence of a suppressed Th1 response comes from the levels of expression of IL-13, the major Th2 cytokine in chickens [28, 36] and the anti-inflammatory IL-10, which were both significantly up-regulated at 11 and 14 dpi. However, it should be noted that mRNA expression levels of the other canonical Th2 cytokine, IL-4, were down-regulated from 7 dpi and those of the other anti-inflammatory cytokine, TGF-β4, were only slightly up-regulated (less than 2-fold) from 11 dpi.

It thus seems that CAV interferes with the induction of both a full innate response, but certainly the appropriate Th1 adaptive response, especially since our gross pathological findings (Table 1) indicated severe viral destruction of the thymus cortex, as has been consistently reported in CAV studies [4, 9, 38]. In the other lymphoid tissues studied, there was no discernible pattern of cytokine regulation, except for up-regulation of IL-2, IL-10 and IL-13 in the bone marrow, mirroring what was seen in the thymus. Microarray analyses have not been previously used to study CAV infection *in vivo*. For our analyses we used an immunologically relevant focused microarray chip created from a pool of stimulated immune tissues containing probes of genes that represent a wide spectrum of immune functions [21]. We believe that a focused array system is preferable to obtain a true transcriptomic representation of a particular immune phenotype even though that possible regulation of genes involved in non-immune pathways may be missed. Apart from the obvious technical and economic advantages, focused arrays may benefit from higher statistical power than universal arrays, by analysis of a considerably smaller dataset and thus eliminating possible masking of data by multiple-testing corrections to the significance levels [39]. The most significant changes in terms of FDR and actual number of modulated genes were observed in thymus, with fewer in the spleen and the bone marrow, confirming again that the primary infection site is the thymus [1, 4, 40]. The lack of gene modulation in the bursa of Fabricius was striking, but not unexpected considering that it is relatively lacking in T-cells, the primary target cells of CAV infection. The subtle magnitude of host gene expression differences identified by both qRT-PCR and microarrays and the stringent filtering during array analysis could account for the disparity found for expression levels of several cytokines between the two assay systems. It is also possible that depleted and abundant T cell populations in CAV- and mock- infected respectively thymuses may be at least partly responsible for the identified microarray transcriptional changes. Nevertheless, the microarray findings were confirmed by qRT-PCR that is normalized against a reference gene to compensate for differences in the mRNA template.

As described earlier, VP3 or “apoptin” induces apoptosis in various human tumour and transformed cells [10, 41, 42]. Apoptin presumably translocates to the nucleus and triggers apoptosis, via a mechanism that remains largely unknown, but possibly by releasing cytochrome c and other pro-apoptotic mitochondrial proteins from mitochondria [43]. Release of these proteins is essentially controlled by Bcl-2 family proteins, which include proteins with both pro- and anti-apoptotic activities [43, 44]. The microarray analyses indicated a marked up-regulation of mRNA expression levels of the pro-apoptotic Bcl-2 family member Bcl2-antagonist/killer 1 (BAK1). This was confirmed by qRT-PCR (Table 5). This result supports previous work showing that apoptin-induced cell death is p53-independent and mediated by members of the Bcl-2 family [41, 45]. Although the exact mechanisms by which Bcl-2 family members regulate apoptosis are not fully understood, pro-apoptotic Bcl-2 proteins can bind and deactivate anti-apoptotic proteins, which normally act to preserve mitochondrial membrane integrity and thus prevent the release of cytochrome c [44, 46].

Immune responses to viral antigens can often be disrupted by intrinsic defects in signalling pathways. Suitable activation and tight regulation of signalling from the TCR are critical for T-cell development in the thymus, as well as for initiating immune responses in mature T-cells [47]. Genes involved in TCR signalling and maturation, such as SLA, GRAP2, the CD8α chain precursor and SATB1, were down-regulated, confirming T-cell dysfunction during CAV infection. The down-regulation of SLA and SATB1 were also confirmed by qRT-PCR (Table 5). SLA is a negative regulator of TCR levels during T-cell development [47] and its down-regulation or absence can lead to alterations in T-cell signalling, development and maturation [48–50]. GRAP2 encodes a protein homologous to human growth factor receptor- binding protein (GRB2), an adaptor protein that is expressed predominantly in T-cells and acts as a positive regulator of multiple TCR signalling pathways, including the CD28 complex [51]. GRB2 is also associated with β-catenin/Wingless (Wnt) protein signalling [52], which plays a major role in the development of T-cells and the transcription of factors that control the expression of genes needed for cell growth [53]. Mutations in GRB2 result in impairment of TCR signalling [51]. Similarly, T-cell surface antigen CD2, an adhesion molecule involved, through its interaction with LFA-3 (CD58), in facilitating CD3/TCR recognition of the antigens presented via the major histocompatibility complex (MHC) and in T-cell activation [54], was down-regulated in infected chickens. SATB1 is expressed primarily in thymocytes and in Th2 cells [55] and is a nuclear protein that binds to the bases of chromatin loop domains and keeps chromatin in place. It is therefore a likely target for degradation in early apoptosis in order to efficiently disassemble chromatin. SATB1 is also a global regulator, including regulation of expression of the Th2 cytokines IL-4, IL-5 and IL- 13 [55–57]. It also interacts with Wnt protein signalling [53]. Its marked down- regulation in this experiment may facilitate or reflect apoptosis induced by CAV. Given the similarities between the regulation of human and chicken TCRs and downstream signalling [58], it is reasonable to assume that down-regulation of these genes may result in deficiencies in TCR signalling in CAV-infected chicken cells. A set of genes involved mainly in cell signalling and transcription, that includes genes with a likely role in facilitating or blocking virus propagation, were also differentially regulated following CAV infection.

Up-regulated transcripts included the transcription factor nuclear factor erythroid 2-like 2 (NFEF2L2) in the thymus, the coatomer protein-complex subunit epsilon (COPE) in the bone marrow and thymus, which is a trafficking protein possibly involved in the transport of viral proteins [59]. In addition, in the spleen, bone marrow and thymus the paracaspase MALT1, a critical mediator of T- and B- cell receptor signalling through cytoplasmic sequestration of Nf-kB and apoptosis [60], was also notably up-regulated.

The mRNA expression levels of several immunoglobulin chain genes were significantly and strikingly up-regulated in the thymus following CAV infection (S2 File). This could be due to an influx of B-cells or activation of B-cells already in the thymus. Upon examination of thymic sections via immunohistochemistry, we found no evidence of a B-cell influx into the thymus (data not shown), suggesting a possible activation of thymus-resident B-cells following CAV infection, perhaps related to the observed up-regulation of IL-10 and IL-13. However, it remains unclear whether the numerically minor thymic-resident B-cells are truly activated during CAV infection and why the B lymphocyte precursors are not affected by CAV.

This study suggests that CAV, like many other viruses obstructs/evades host cellular anti-viral processes and exploits the host cellular resources to produce viral gene products. The microarray analyses presented here confirm previous studies that apoptosis occurs following CAV infection [1, 12, 61], involving the pro-apoptotic Bcl-2 family member Bcl2-antagonist/killer 1 (BAK1). The combined qRT-PCR and microarray analyses suggest that CAV mediates immunosuppression by suppressing induced innate (pro-inflammatory) and subsequent adaptive responses in T-cells. Virus-induced dysregulation of TCR signalling may also provide a strategic mechanism for virus escape from the immune system. Parallels occur in other viruses such as measles virus [62], herpes simplex virus [63] and human T-cell leukaemia/lymphoma virus type 1 [64], which all inhibit or deregulate TCR signalling. Our data provide novel information on key genes to understand CAV pathogenicity and also functional pathway networks that may inform vaccine strategies. The exact mechanisms by which the viral proteins manipulate the TCR and other host signalling pathways, and drive the onset of pro-apoptotic events, remain to be elucidated.

## Supporting Information

S1 File Ingenuity pathway analysis. File contains information about: Ingenuity canonical pathways, biological functions and networks; *p*-Value, score and number of genes (molecules) implicated in each category.
(XLS)

S2 File Full list of genes differentially regulated between control and CAV-infected birds at 14 dpi in thymus, bone marrow and spleen. (XLS)
